# Association-sensory spatiotemporal hierarchy and functional gradient-regularised recurrent neural network with implications for schizophrenia

**DOI:** 10.1038/s41540-026-00727-x

**Published:** 2026-04-30

**Authors:** Subati Abulikemu, Puria Radmard, Michail Mamalakis, John Suckling

**Affiliations:** 1 Department of Psychiatry, University of Cambridge, Cambridge, UK; 2 Centre for Human-Inspired Artificial Intelligence, University of Cambridge, Cambridge, UK; 3 Department of Engineering, University of Cambridge, Cambridge, UK; 4 Department of Computer Science and Technology, University of Cambridge, Cambridge, UK

**Keywords:** Computational biology and bioinformatics, Neuroscience

## Abstract

The human neocortex is functionally organised at its highest level along a continuous sensory-to-association (AS) hierarchy. This study investigates two questions—how this hierarchy is structurally altered in schizophrenia, and what these alterations imply for neural dynamics and cognitive computation. Using a large fMRI dataset (*N* = 355), we extracted individual AS gradients via spectral analysis of brain connectivity and quantified hierarchical organisation by the gradient range. Schizophrenia showed a compressed AS hierarchy, indicating reduced functional differentiation. Estimating neural timescale (autocorrelation decay constant) with the Ornstein-Uhlenbeck process, we observed that the most specialised, locally cohesive regions at the gradient extremes exhibit longer timescales, an empirical spatiotemporal mapping that is attenuated in schizophrenia. To probe the computational consequences of this compression, we used the gradients to regularise subject-specific recurrent neural networks (RNNs) trained on working memory tasks. Networks endowed with greater gradient range learned more efficiently, plateaued at lower task loss, and maintained stronger alignment to the prescribed AS hierarchical geometry. Fixed-point linearisation showed that high-range networks settled into more stable neural states during memory delay, evidenced by lower energy and smaller maximal Jacobian eigenvalues. This gradient-regularised RNN framework thereby links large-scale cortical architecture with fixed point stability, providing a computational hypothesis that AS gradient de-differentiation can destabilise neural computations in schizophrenia, convergently supported by empirical timescale flattening along AS gradient and model-based evidence of less stable fixed points.

## Introduction

The human neocortex operates through coordinated, hierarchically organised modules that support both integrative and specialised functions. In schizophrenia, this architecture is posited to be disrupted by dysconnectivity—aberrant connections among neural ensembles that impair information processing^1–3^. Recent cortical mapping revealed a continuous hierarchy extending from primary sensory systems to transmodal association networks, evident across scales from gene expression and cytoarchitecture to morphology and macroscale functional connectivity (FC)^4–8^. One way to characterise this hierarchy is through FC gradients, which provide a low-dimensional coordinate system derived from the similarity structure of FC patterns across regions. The principal association-sensory (AS) functional gradient delineates a smooth, computationally meaningful continuum from perception to abstract cognition^9^. This study has two aims within this gradient framework. First, we experimentally examine how the AS gradient and its relationship with neural dynamics are altered in schizophrenia. Second, we ask what changes in AS's hierarchical organisation imply for cognitive computation, by embedding empirically derived gradients as architectural constraints in recurrent neural networks (RNNs) trained on cognitive tasks.

Functional dysconnectivity holds promise as an explanatory framework for schizophrenia, supported by extensive fMRI evidence of abnormal FC, reduced small-worldness, and diminished functional segregation^10–12^. However, findings at the level of individual regions remain inconsistent, reflecting the idiosyncratic nature of brain organisation compounded by heterogeneity in cortical mapping strategies. Because FC gradients are computed from the same FC matrix that underlies edge-wise and graph-theoretic measures, they are not independent of conventional connectomic features; rather, they provide a complementary description that summarises dominant, continuous axes of system-level organisation^13^. From a computational standpoint, capturing such global hierarchical organisation may offer a basis for modelling how network architecture shapes information diffusion, neural dynamics stability, and cognition. In this context, recent studies have reported compression or functional de-differentiation of the AS gradient in schizophrenia^14–16^. Addressing the first aim, we begin by examining the relationship between the spatial AS gradient and the brain’s temporal organisation, as a means of characterising how this compression may manifest in neural dynamics.

The hierarchical structure inherent in the neocortex’s functional architecture is likely mirrored in its dynamics, specifically in neural timescales, defined as the decay time constant of the autocorrelation function of spontaneous activity^17,18^. Multimodal evidence suggests a global hierarchy of temporal integration windows, indexed by signal autocorrelation decay, which lengthens from early sensory to higher-order areas^17,19–22^. Within this global trend, more granular system-specific temporal gradients have been indicated^23,24^. However, the relationship between the AS gradient and temporal integration may not be strictly monotonic, considering the modular organisation of neural systems and evidence connecting longer timescales to greater within-community FC^25^. Under the gradient framework, AS gradient extremes encode highly specialised nodes with functionally similar neighbours. Consequently, protraction toward either extreme of the AS gradient likely reflects increasing within-subsystem integration. We therefore hypothesise that the spatiotemporal mapping reflects nested timescale hierarchies within each subsystem, a structure we predict to be flattened in schizophrenia.

This prediction is motivated by the neurodynamical hypothesis of schizophrenia, which proposes that brain network activity is destabilised by shallow attractor states^26,27^. These attractors represent stable patterns of neural activity that underpin cognitive processes such as working memory. Computationally, instability implies that neural networks fail to robustly maintain these patterns and are easily perturbed, causing sudden and more frequent transitions across states. This fragility aligns with a dampening of neural integration windows in schizophrenia, leading to temporal fragmentation in neural coding. Indeed, resting-state fMRI studies have evinced brain-level timescale reductions in schizophrenia relative to controls^23,28^. Alterations in the intrinsic relationship between timescales and the AS gradient may help contextualise dysconnectivity in schizophrenia, and how it translates to characteristic effects on cognition.

Addressing the second aim, moving beyond empirical associations to understand how gradient disruptions influence cognitive computations necessitates computationally explicit modelling. To this end, RNNs serve as powerful means for generating and testing mechanistic hypotheses of neural computations^29–34^. When task-optimised RNNs are trained on the same behavioural paradigms used during neural recordings, they can reproduce observed population dynamics and reveal previously unknown computational mechanisms^31^. The mapping from network architecture to behaviour is typically many-to-one, such that substantially different connectivity patterns could achieve comparable performance, thereby highlighting the role of regularisations in the optimisation process^29,35^. In a complementary line of research, empirical connectivity can be embedded as biologically informed organisational constraints on the network during training^36–39^. From the resulting nonlinear dynamical systems, one can examine how attractor-like, slow regions in the state space implement cognitive computations; for instance, through linearisation analyses of emergent dynamical motifs^33,40^. Under this framework, we explicitly probe whether AS gradient compression, as hypothesised in schizophrenia, degrades the computational and dynamical properties of gradient-regularised RNNs trained on cognitive tasks.

Taken together, this study integrates experimental findings from resting-state fMRI with theoretical models to elucidate the implications of the degree of differentiation along the AS gradient—quantified as the gradient range—for neural dynamics and computation. On the empirical side, we extracted the AS gradient and assessed its intrinsic mapping to neural timescales, with a particular focus on alterations in schizophrenia. We sought to replicate previously reported AS gradient de-differentiation and test whether schizophrenia exhibits a dampened, more homogeneous distribution of neural integration windows, with diminished relative slowness in specialised, locally cohesive communities. On the computational side, we developed a generative framework that embeds the empirically derived AS gradient as connectivity constraints in RNNs, leveraging the link between gradient range and connectivity weight geometry. We specifically focused on the AS gradient as it encapsulates the most global hierarchical specialisation, covering both lower- and higher-order systems. By regularising the working memory-performing RNNs with AS connectivity matrices generated from empirical AS gradients, we directly assessed how variations in gradient range modulate network learning and dynamical stability.

## Results

### Principal cortical gradient and its reorganisation in schizophrenia

**Fig. 1:**
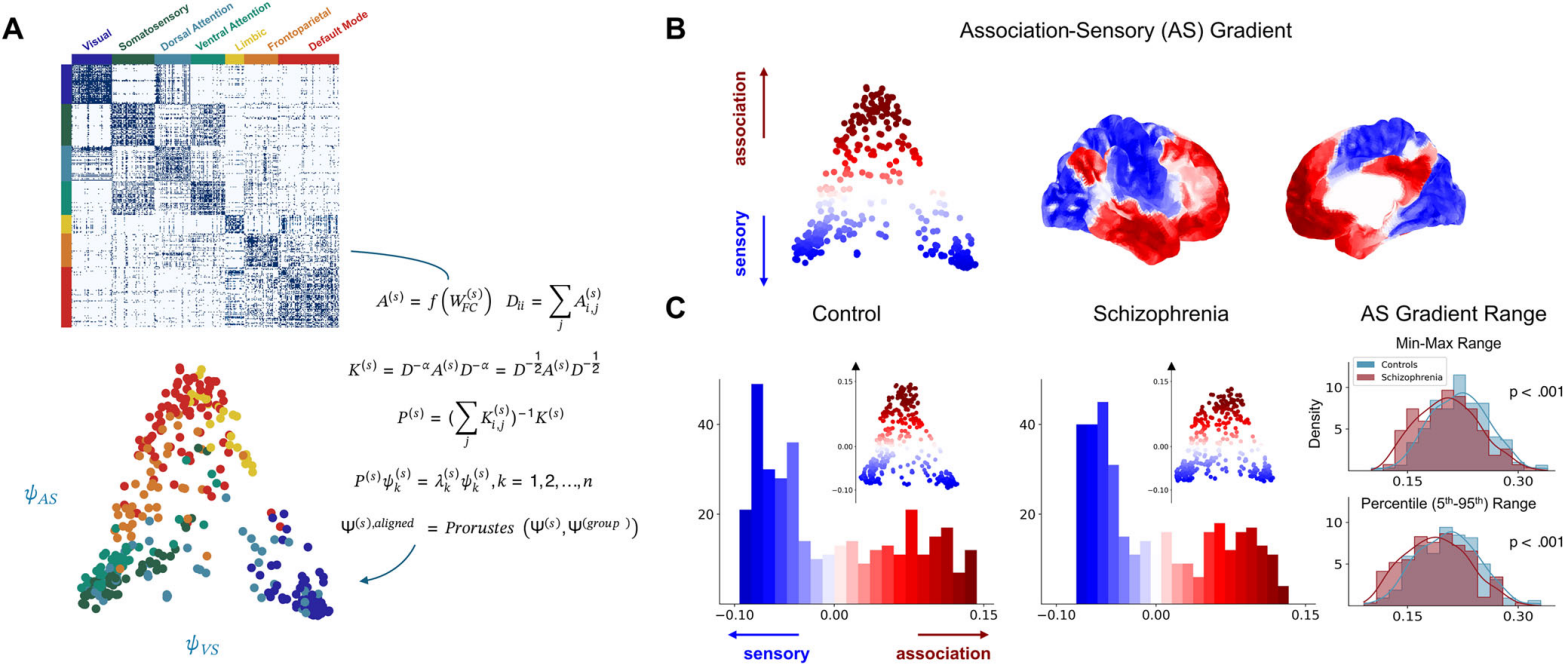
Compressed association-sensory gradient in schizophrenia. **A** Diffusion map embedding was applied to the sparsified and similarity-transformed FC matrix, producing a spectral decomposition of the similarity matrix. The two primary gradients are the AS gradient (*ψ*_*AS*_) and the unimodal gradient differentiating sensory modalities (*ψ*_*VS*_). **B** Cortical projection of the principal AS gradient illustrates the continuous organisation from unimodal (blue) to transmodal regions (red). **C** The approximate bimodal distribution of the AS gradient values shows a significant compression in schizophrenia.

We analysed resting-state functional MRI (fMRI) data from the Bipolar and Schizophrenia Network for Intermediate Phenotypes (BSNIP) consortium, comprising 186 healthy controls and 169 schizophrenia proband patients across four study sites (Baltimore *N* = 132, Hartford *N* = 92, Dallas *N* = 81, and Boston *N* = 43)^41,42^. Our goal was to investigate whether schizophrenia involves disruptions in hierarchical functional organisation. To this end, we decomposed the sparsified and similarity-transformed functional connectivity (FC) matrices through diffusion map embedding (Fig. 1A, B; see “Methods” subsection “Spatial gradients via diffusion map”). We specifically focused on the principal association-sensory (AS) gradient, *ψ*_*AS*_—the lowest-frequency (most global) eigenmode capturing the unimodal sensory to transmodal association continuum. The following results were consistent across different network density thresholds applied at sparsification (Supplementary Fig. 1); the reported results are based on retaining 50 edges per node (14% of connections per region given 360 cortical regions; see “Methods” subsection “Spatial gradients via diffusion map”). All subsequent regression analyses use *z*-scored continuous variables.

The information explanatory power of *ψ*_*AS*_ was significantly lower in schizophrenia patients (mean eigenvalue ratio = 0.19) than in healthy controls (mean eigenvalue ratio = 0.21), after adjusting for age, gender, site, and TR using multiple regression analysis (*β* = −0.45, *p* < 0.001, 95% CI = [−0.66, −0.24], Cohen’s *d* (unadjusted) = −0.46). A critical focus of this study was the gradient range, indexing the extent of FC differentiation and hierarchical organisation along the eigenmode. Larger range of *ψ*_*AS*_ (range_AS_) signifies stronger separation between sensory- and association-like connectivity patterns (more differentiated hierarchy), while lower range_AS_ indicates a compressed hierarchy. Using the same regression approach, we observed a significantly lower range_AS_ in schizophrenia patients (mean range_AS_ = 0.20) compared to healthy controls (mean range_AS_ = 0.22; *β* = −0.35, *p* < 0.001, CI = [−0.55, −0.14], Cohen’s *d* = −0.39; Fig. 1C).

To assess whether this group difference could be driven by global FC strength or by sensitivity of the min-max range definition to extreme parcels, we performed two robustness checks. The effect persisted after additionally controlling for mean nodal FC strength (from sparsified matrices; *β* = −0.37, *p* < 0.001, CI = [−0.58, −0.17]) and when defining AS dispersion using the 5th–95th percentile range (*β* = −0.36, *p* < 0.001, CI = [−0.56, −0.15]). Thus, functional differentiation along the AS hierarchy appears attenuated in schizophrenia, with effect sizes in the small to moderate range. By contrast, the range of the second (visual-somatosensory) gradient showed no evidence of a group difference (*β* = 0.005, *p* = 0.17, CI = [−0.002, 0.013]), suggesting the compression is specific to the principal AS axis.

To evaluate the behavioural relevance of the altered *ψ*_AS_ gradient range, we examined its association with cognitive function measured by the composite Brief Assessment of Cognition in Schizophrenia (BACS), while accounting for diagnosis and the confounding variables above. Regression analyses revealed that range_AS_ was positively associated with cognition (*β* = 0.11, *p* = 0.03, CI = [0.01, 0.21]; *N*_HC_ = 151, *N*_SZ_ = 151) across all samples with a small effect size. However, this association was not robust within the schizophrenia cohort alone (*β* = 0.14, *p* = 0.09, CI = [−0.02, 0.29]). Within the schizophrenia cohort alone, ${{{\rm{range}}}}_{{{\rm{AS}}}}$ showed a negative association with positive symptom severity (PANSS Positive Scale; *β* = −0.19, *p* = 0.02, CI = [−0.35, −0.03]; *N*_SZ_ = 159), but no significant relationship with negative symptom severity (*β* = −0.06, *p* = 0.52, CI = [−0.23, 0.12]).

### Association-sensory gradient range and network specialisation

Having observed the compressed AS gradient in schizophrenia, we next asked what this compression reflects in the underlying connectivity structure. Since *ψ*_AS_ is computed from FC, this section provides a descriptive characterisation of how *ψ*_AS_ range (range_AS_) is reflected in the weight geometry of the FC matrix (*W*_FC_), rather than an independent test. Specifically, we aimed to (1) clarify how variations in range_AS_ correspond to distinct near-to-distant (in *ψ*_AS_ space) connectivity profiles in *W*_FC_, and (2) establish an empirical basis for constructing the subject-specific association-sensory weight matrix (*W*_AS_) used in the subsequent theoretical analysis.

**Fig. 2:**
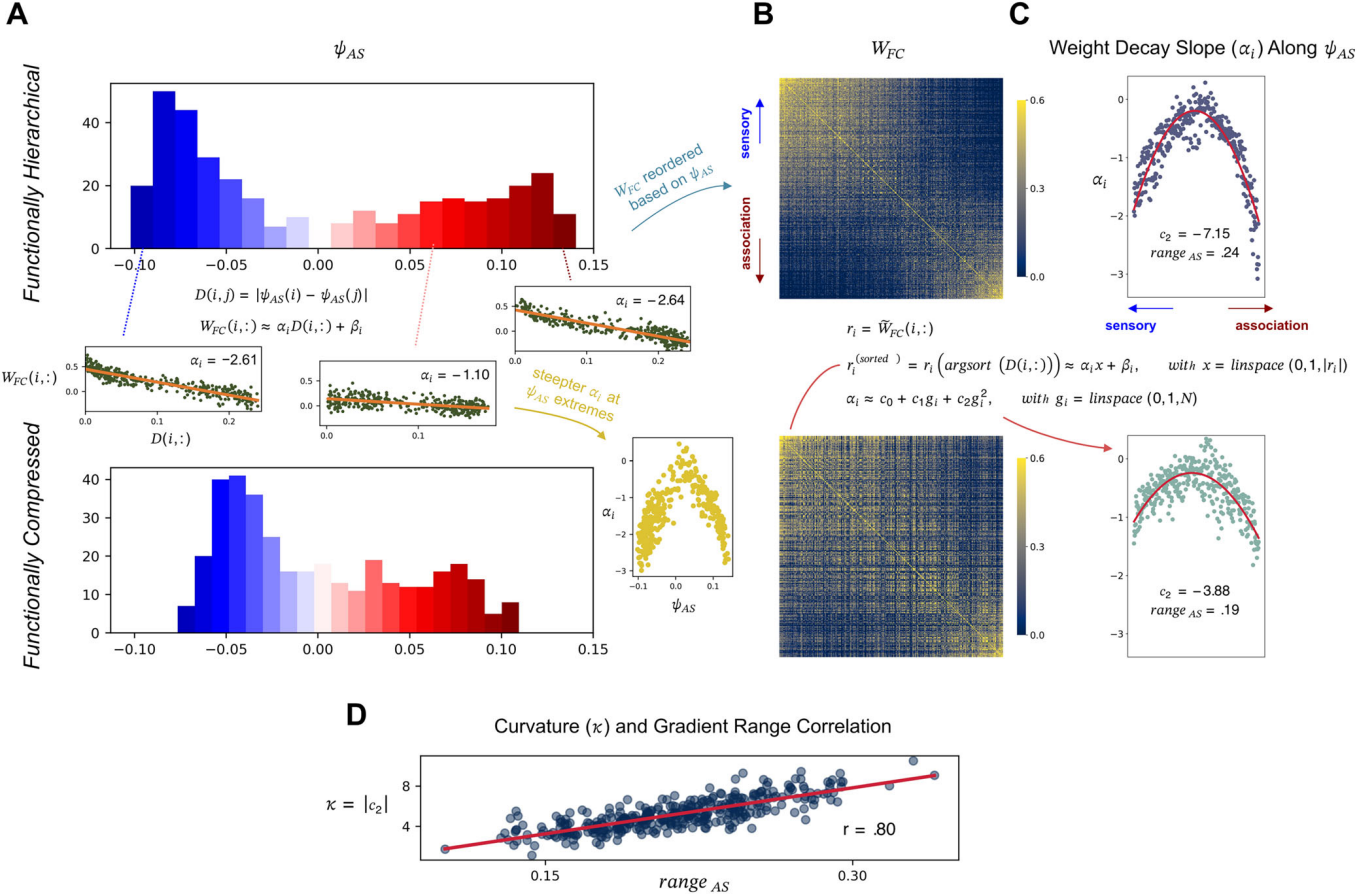
Gradient range and weight geometry. **A** The association-sensory gradient (*ψ*_AS_) distributions for a functionally hierarchical, high-range network (top) and functionally collapsed, low-range network (bottom). Connectivity weights between a given node and others decline with increasing gradient distance (middle). Nodes closer to sensory or associative poles exhibit steeper connectivity decay, indicating greater local specialisation. **B** Connectivity matrices (*W*_FC_) reordered based on regional positions on the *ψ*_AS_ gradient for a high-range (top) and low-range network (bottom). After *z*-scoring at the matrix level, each connectivity vector (row) was sorted by gradient distance and fitted using a linear model, resulting in a vector of slope values $\alpha =[{\alpha }_{1},{\alpha }_{2},\ldots {\alpha }_{N}]$ (middle). **C** The node-wise slopes (${\alpha }_{i}$) were further modelled by a quadratic function with normalised gradient positions along *ψ*_AS_ as the independent variable. The curvature magnitude $\kappa =\left|{c}_{2}\right|$ quantifies the extent to which local-distant contrast is exhibited by the association and sensory extremes, hence a more functionally differentiated (hierarchical) organisation. **D** Strong positive alignment between curvature ($\kappa$) and AS gradient range (range_AS_; 60% of the strongest connections retained per node for linear models).

For each subject, AS gradient distance between regions *i* and *j* was defined as ${D}_{{ij}}=\left|{\psi }_{{{\rm{AS}}}}\left(i\right)-{\psi }_{{{\rm{AS}}}}\left(j\right)\right|$. For a given node *i*, its connectivity weights *W*_FC_ (*i*, :) systematically weaken with increasing ${D}_{i:}$, a relationship that can be summarised with a simple linear trend between weights and gradient distance. Empirically, this distance-weight slope is more negative near the gradient extremes, reflecting enhanced local specialisation proximal to sensory and associative poles (Fig. 2A, middle panels). This decay geometry along ${\psi }_{{{\rm{AS}}}}$ provides the empirical motivation for the structure of *W*_AS_ introduced later.

To explicitly quantify how range_AS_ reflects network specialisation in connectivity space, we fitted linear models to the rows of connectivity matrices. Each row, denoted as ${r}_{i}={\widetilde{W}}_{{{\rm{FC}}}}(i,:)$, where ${\widetilde{W}}_{{{\rm{FC}}}}$ is the within-subject *z*-scored FC matrix, was sorted by increasing gradient distance (${D}_{i:}$) and modelled as:

1 $${r}_{i}^{({{{sorted}}})}\approx {\alpha }_{i}x+{\beta }_{i}$$

The regressor $x\in [\mathrm{0,1}]$ is the normalised rank index after sorting the $M$ retained nodes by distance (i.e., $x=(m-1)/(M-1)$, where $m$ is the position in the sorted list), such that $x=0$ corresponds to the node closest to *i* in *ψ*_AS_ space and $x=1$ to the most distant; using rank-normalised $x$ standardises the near-to-far profile across subjects with different ${\psi }_{{{\rm{AS}}}}$ scales. The slope *α*_*i*_, therefore, quantified the node *i* ’s local-distant connectivity contrast along the AS axis (more negative *α*_*i*_ indicates steeper decay).

We summarised this pattern across nodes by relating ${\alpha }_{i}$ to each node’s normalised position along the AS axis. Specifically, after ordering nodes by *ψ*_AS_ (sensory to association), we defined ${g}_{i}=({{\rm{rank}}}(i)-1)/(N-1)\in [\mathrm{0,1}]$ (from most sensory = 0 to most association = 1), and fitted a quadratic function

2 $${\alpha }_{i}\approx {c}_{0}+{c}_{1}{g}_{i}+{c}_{2}{g}_{i}^{2}$$

We used $\kappa =\left|{c}_{2}\right|$ as a curvature summary of how strongly local-distant contrast is concentrated towards the association and sensory extremes (Fig. 2B, C). Across subjects, $\kappa$ strongly correlated with range_AS_ (Fig. 2D) and remained robust ($r=0.77-0.80$) across connectivity thresholds (retaining the top 50–80% of connections per node).

Networks with larger range_AS_ showed sharper pole-centred local-distant contrast along *ψ*_AS_, whereas compressed gradients were associated with more homogenous profiles.

### Neural timescale estimation and spatial-temporal convergence

Beyond the spatial compression of the AS gradient, we asked whether this reorganisation is empirically reflected in the temporal domain. Here, we investigated whether the intrinsic neural timescales of cortical regions are related to their position along *ψ*_AS_, without imposing a rigid sensory-association dichotomy, and whether this spatiotemporal relationship is altered in schizophrenia. We defined the neural timescale as the time constant of the exponential decay of the signal autocorrelation function (ACF). To do so, we first evaluated timescale estimation via simulation, comparing a maximum likelihood estimate (MLE) framework based on the Ornstein-Uhlenbeck (OU) process to the typical direct exponential fitting of the sample ACF, which risks systematic bias under finite time series lengths (see “Methods” subsection “Neural timescale estimation”)^43–45^.

To validate the OU-MLE approach, we simulated univariate OU time series with a ground-truth timescale, *τ*, of 3 s, 5 s, and 10 s, spanning preliminary empirical estimates from random selection of participants. We varied the sampling interval ∆*t* from 1 s to 5 s in 0.5 s increments and the number of time points from 100 to 500 in increments of 50, covering typical fMRI settings. For each parameter combination, we generated 100 random time series instances and fitted them using both methods.

**Fig. 3:**
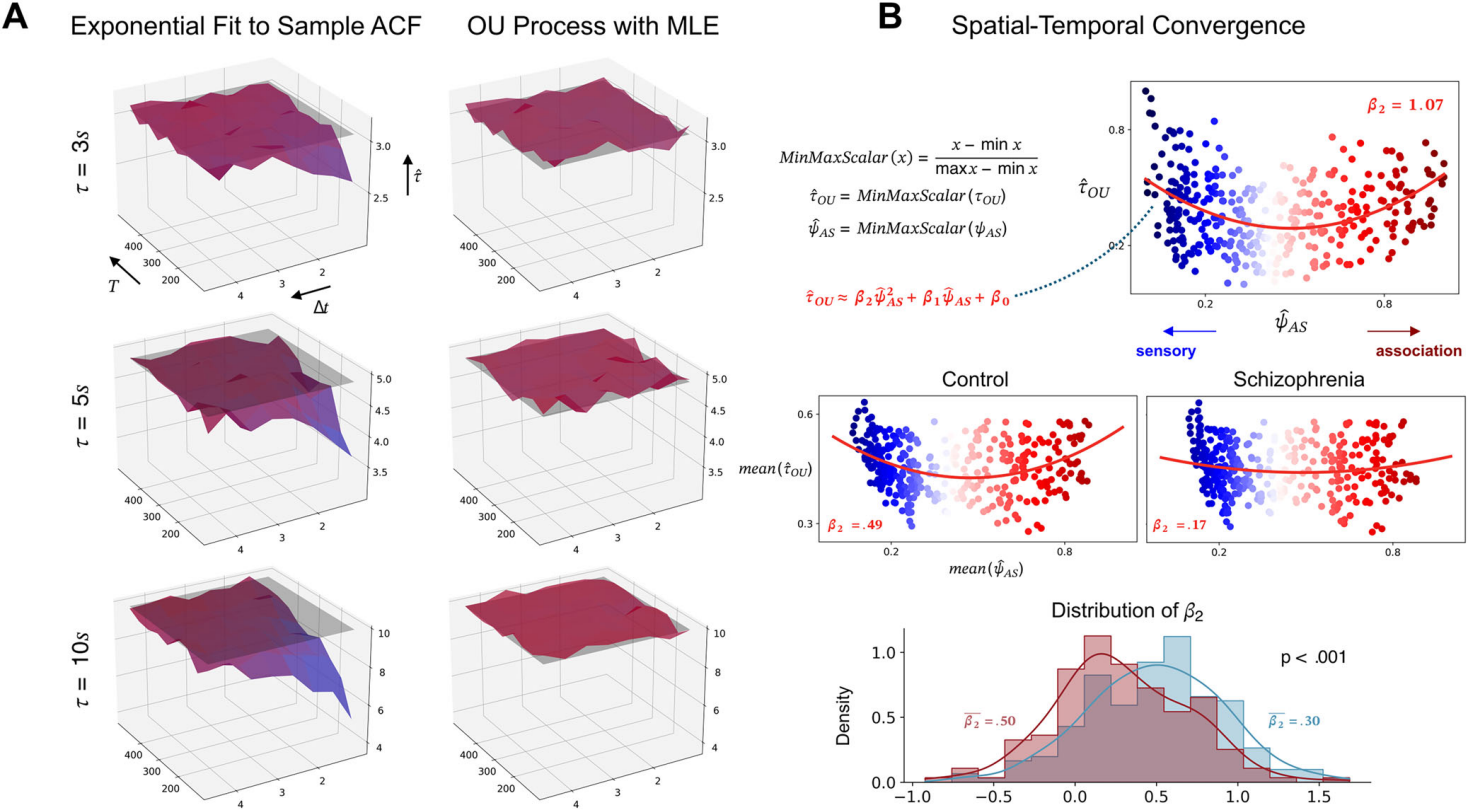
Neural timescale estimation using an Ornstein-Uhlenbeck process (OU) with maximum likelihood (MLE). **A** The timescale estimation with ground truth *τ* of 3 s, 5 s, and 10 s using direct-exponential (left) and OU-MLE (right); for direct-exponential, the maximal time lag *l*_max_ was set to half of the sample length. Biased $\hat{\tau }$ with the conventional direct-exponential approach was particularly pronounced at low ∆*t* (1–3 s) and short *T* (100–300 frames). **B** The intrinsic relationship between within-subject Min-Max normalised *ψ*_*AS*_ and *τ*_*OU*_ modelled with a quadratic function in an example participant (top), and group-averaged representations for both groups (middle). The distribution of quadratic coefficient *β*_*2*_ and group comparison via multiple regression revealed a significantly dampened *τ*_*OU*_ -*ψ*_*AS*_ relationship in schizophrenia.

The simulation confirmed the superiority of OU-MLE over direct exponential fitting (Fig. 3A), as also seen by Strey (2019)^44^. Under a true *τ* of 3 s, 5 s, and 10 s, OU-MLE estimates yielded means $\hat{\tau }$ of 3.02 s (mean absolute error mae = 0.41, variance var = 0.29), 5.02 s (mae = 0.70, var = 0.86), and 10.04 s (mae = 1.70, var = 5.31), respectively. The direct exponential fit returned means $\hat{\tau }$ of 2.93 (mae = 0.51, var = 0.47), 4.80 s (mae = 0.99, var = 1.64), and 9.16 s (mae = 2.53, var = 9.55). The exponential fit exhibited *τ* underestimation, particularly at low ∆*t* (1–3 s) and short *T* (100–300 frames), conditions that are especially relevant for empirical fMRI samples. In contrast, the OU-MLE approach remained more robust, with lower estimation error and variance across conditions.

Having validated OU-MLE, we next examined whether the variation along *ψ*_*AS*_ aligns more closely with a simple linear relationship to *τ*, i.e., lengthening of timescale along the sensory-to-association gradient, or whether progressive specialisation toward the gradient extremes gives rise to a hierarchical temporal structure within both sensory and associative systems. Using the OU-MLE derived timescales *τ*_*OU*_, we compared linear vs. quadratic models of *τ*_OU_-*ψ*_AS_ (both Min-Max normalised). A paired-sample *t*-test of Akaike Information Criterion (AIC) showed the quadratic model fit significantly better than the linear model (mean ∆AIC = −17.8, *p* < 0.001). Under the quadratic model, schizophrenia patients exhibited smaller quadratic coefficients *β*_2_ (mean *β*_2_ = 0.29 vs. 0.50) after adjusting for age, gender, site, and TR (Fig. 3B; normalised *β* = −0.38, *p* < 0.001, 95% CI = [−0.58, −0.17], Cohen’s *d* (unadjusted) = −0.48). This indicates a moderately more pronounced U-shaped *τ*_OU_-*ψ*_AS_ relationship for controls relative to the flatter curve observed in schizophrenia patients, suggesting diminished temporal differentiation at the gradient extremes (i.e., relative reduction in the slowing of dynamics within the most strongly associative and sensory regions).

### Association-sensory differentiation and recurrent network dynamics

**Fig. 4:**
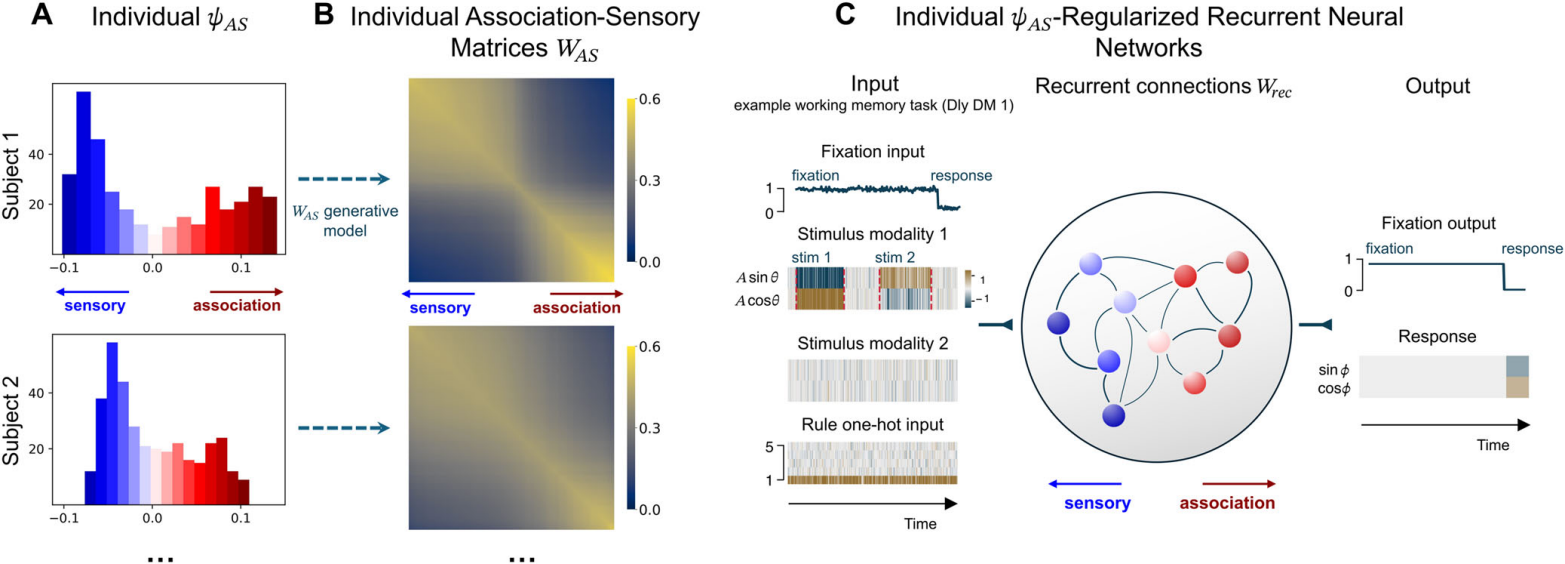
Pipeline for the theoretical studies. **A** Subject-specific gradients *ψ*_AS_. **B** Subject-specific AS matrices *W*_AS_ derived from *ψ*_AS_ with the generative model (“Methods” subsection “Generative model of association-sensory matrix”). **C** For each *ψ*_AS_-*W*_AS_ pair, we train a separate RNN. Five WM variants require the network to remember two sequential stimuli and respond in the direction of the stronger one (i.e., higher-amplitude; “Methods” subsection “Recurrent neural network, working memory tasks, and regularisations”). Inputs (noisy; 10 dim): fixation (1), stimulus modality 1 (sin *θ*, cos *θ* × amplitude; 2), stimulus modality 2 (2), rule one-hot (5). Depending on the task variant, either one or both modalities are present. Output (3 dim): fixation scalar and [sin ϕ, cos ϕ] of the chosen stimulus.

In our theoretical studies, we asked how differences in the association-sensory (AS) system differentiation, represented by AS gradient range (range_AS_), influence network computation and dynamical stability using subject-specific recurrent neural networks (RNNs). Motivated by the empirical gradient-connectivity geometry established above, we first converted each subject’s empirical gradient *ψ*_AS_ (Fig. 4A) into an AS constraint matrix *W*_AS_ (Fig. 4B) with a generative model. We then trained a separate RNN per participant, with recurrent connectivity regularised toward *W*_AS_, inputs channelled into sensory units, and predictions read out from association units, while learning working memory (WM) tasks. Briefly, the network has 10D noisy inputs (fixation, two stimulus modalities encoded as [sin *θ*, cos *θ*] with amplitude, and a rule cue) and a 3D output (fixation and [sin ϕ, cos ϕ] of the chosen stimulus; Fig. 4C; “Methods” subsection “Recurrent neural network, working memory tasks, and regularisations”), We then related range_AS_ to WM learning and the stability of the underlying dynamics.

### Generative model of association-sensory weight matrix from ψAS embeddings

As illustrated in the overview in Fig. 4, we first construct each participant’s AS connectivity matrix entirely from their empirical gradient *ψ*_AS_, retaining AS-axis structure while excluding all other spectral components. We require a robust correspondence between the empirical *ψ*_AS_ and the principal gradient *ψ*_AS_’ extracted from *W*_AS_. The resulting *W*_AS_ then serves as the recurrent-weight constraint in RNN training (“Methods” subsection “Recurrent neural network, working memory tasks, and regularisations”). Because we *z*-score *W*_AS_ for RNN regularisation, we consistently extract *ψ*_AS_’ from the same standardised *W*_AS_ to maintain methodological alignment.

**Fig. 5:**
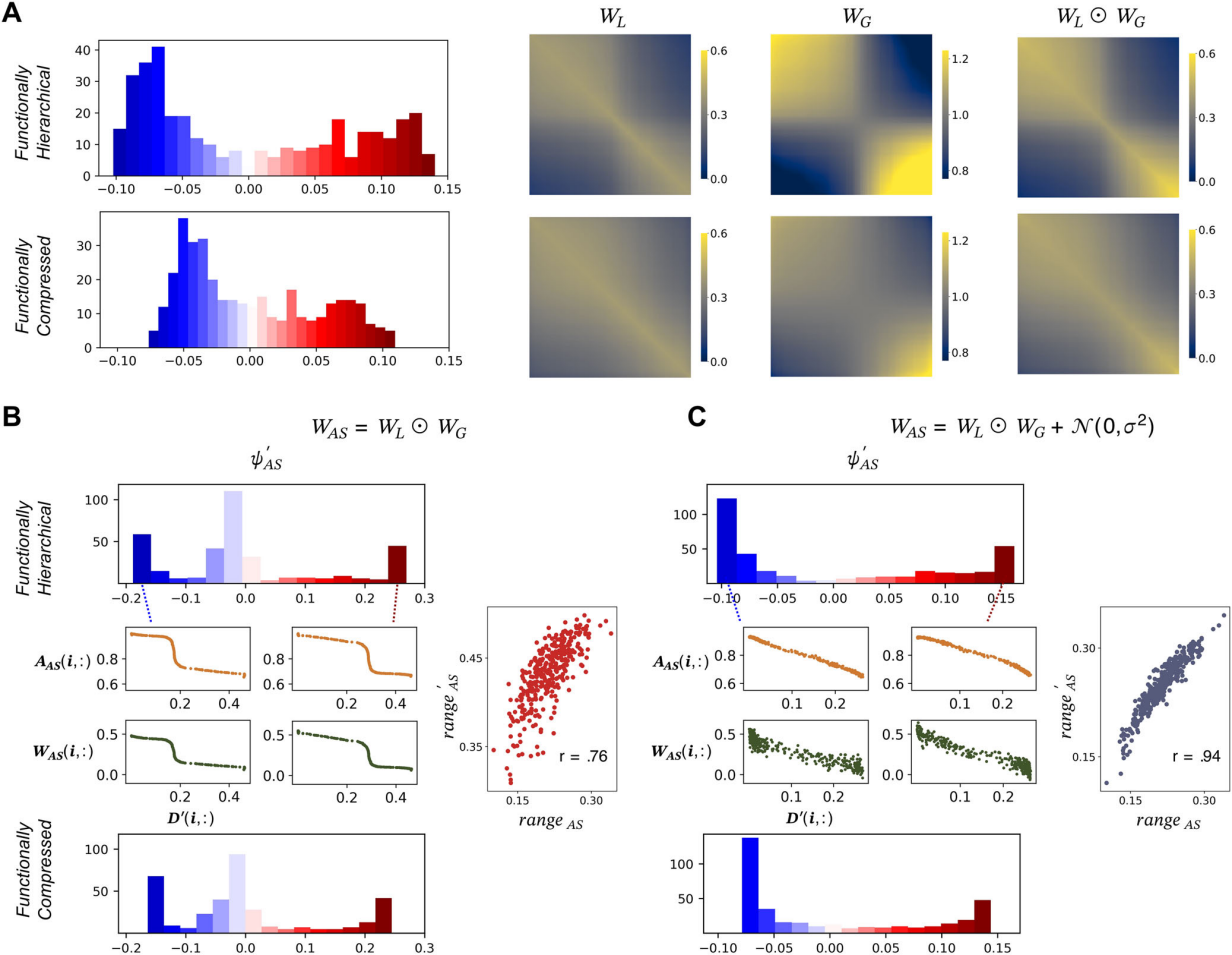
Association-sensory matrix (*W*_AS_) generation. **A** Distributions of empirical association-sensory gradient values colour-coded from sensory (blue) to association (red). On the right are three different matrix constructions, (1) a locality matrix *W*_*L*_ modelling connectivity decay with gradient distance using a node-invariant slope; (2) a global matrix *W*_*G*_ based on scaled outer product of the gradient; and (3) their elementwise product ${W}_{L}\odot {W}_{G}$, for a functionally hierarchical (broader range_AS_; top) and collapsed (reduced range_AS_; bottom) networks. **B** The principal gradient derived from ${W}_{{{\rm{AS}}}}={W}_{L}\odot {W}_{G}$. Across subjects, the recovered gradient range (range_AS_’) correlated at 0.76 with the empirical range_AS_. The deterministic form showed a large intermediate grouping along the sensory (blue)-association (red) axis (top and bottom), with abrupt changes in connectivity similarity and weight with increasing separation along *ψ*_AS_’ (middle). **C** The principal gradient computed when variability was introduced—${W}_{{{\rm{AS}}}}={W}_{L}\odot {W}_{G}+{\mathscr{N}}(0,{\sigma }^{2})$, with range_AS_–range_AS_’ correlation improved to 0.94 and a smoother sensory-association gradient structure.

We tested three distinct approaches for simulating *W*_AS_ (see “Methods” subsection “Generative model of association-sensory matrix”; Fig. 5A):

1. A purely distance-based locality matrix *W*_*L*_, where distance is defined in the *ψ*_AS_ space, so weights decrease with increasing separation along AS axis;
2. A naïve outer product ${\psi }_{{{\rm{AS}}}}{\psi }_{{{\rm{AS}}}}^{T}$; and
3. The integrated form ${W}_{L}\odot {W}_{G}$ that combines local distance-decay and hierarchical scaling (a pole-amplification term) that upweights within-pole (same-sign *ψ*) interactions and downweighs cross-pole (opposite-sign *ψ*) interactions, thereby encoding the empirically observed steeper local-distant contrast at gradient extremes.

Our primary metrics were the correlation between empirical and recovered gradient ranges (range_AS_ vs. range_AS_’), assessing cross-subject gradient range fidelity; and the mean *ψ*_AS_–*ψ*_AS_’ correlations, assessing within-subject preservation of nodal hierarchical orders. The combined ${W}_{L}\odot {W}_{G}$ yielded correlations of 0.76 and 0.92, respectively, surpassing *W*_*L*_ (0.52 and 0.89) and the outer product (0.10 and 0.86). It correlated more strongly with the original connectivity matrix *W*_FC_ (0.43), explaining 0.20 of the variance compared to 0.18 or 0.16 from the other methods.

Despite the robust correlations, the deterministic construction of *W*_AS_ did not sufficiently capture the intended bimodality, instead producing a disproportionally large intermediate grouping between the sensory and association poles (Fig. 5B top and bottom). The effect emerged from applying the same fixed-density threshold (50 connections per node) used for empirical gradients to a smoothly varying deterministic *W*_AS_. Pole regions retained connections almost exclusively to their nearest gradient neighbours, while intermediate nodes accumulated into a large undifferentiated cluster. Introducing controlled variability would therefore help preserve a more continuous, global similarity pattern.

Indeed, injecting a low magnitude of Gaussian noise mitigated these threshold-induced abrupt boundaries and reinforced the alignment with empirical *ψ*_AS_ (Fig. 5C). Averaged over 100 random realisations, the range_AS_–range_AS_’ and mean *ψ*_AS_–*ψ*_AS_’ correlations increased to 0.94 (std = 0.001) and 0.98 (std < 0.001), respectively. Of note, the alignment of *ψ*_AS_’ from *z*-scored and original *W*_AS_ under both metrics was ≈1. Thus, coupling local-global generation with controlled stochasticity enabled a construction of the association-sensory constraint matrix *W*_AS_ whose principal gradient aligns closely with the empirical *ψ*_AS_.

### Recurrent neural network and working memory task learning dynamics

Having generated *W*_AS_ from *ψ*_AS_, we next assessed the computational and dynamical consequences of varying *ψ*_AS_ range. To this end, we trained one continuous-time RNN per subject-specific *W*_AS_–*ψ*_AS_ pair on a family of parametric working memory (WM) tasks and constrained its recurrent weights toward the corresponding *W*_AS_ (Fig. 4). We implemented an RNN architecture and learning protocol similar to previously studied multitask frameworks (detailed in “Methods” subsection ”Recurrent neural network, working memory tasks, and regularisations”)^32,33^. Each WM trial presents two brief stimuli (circular variables with both angle and amplitude), separated by delays, and the network is required to report the stronger stimulus^32,33,46^.

**Fig. 6:**
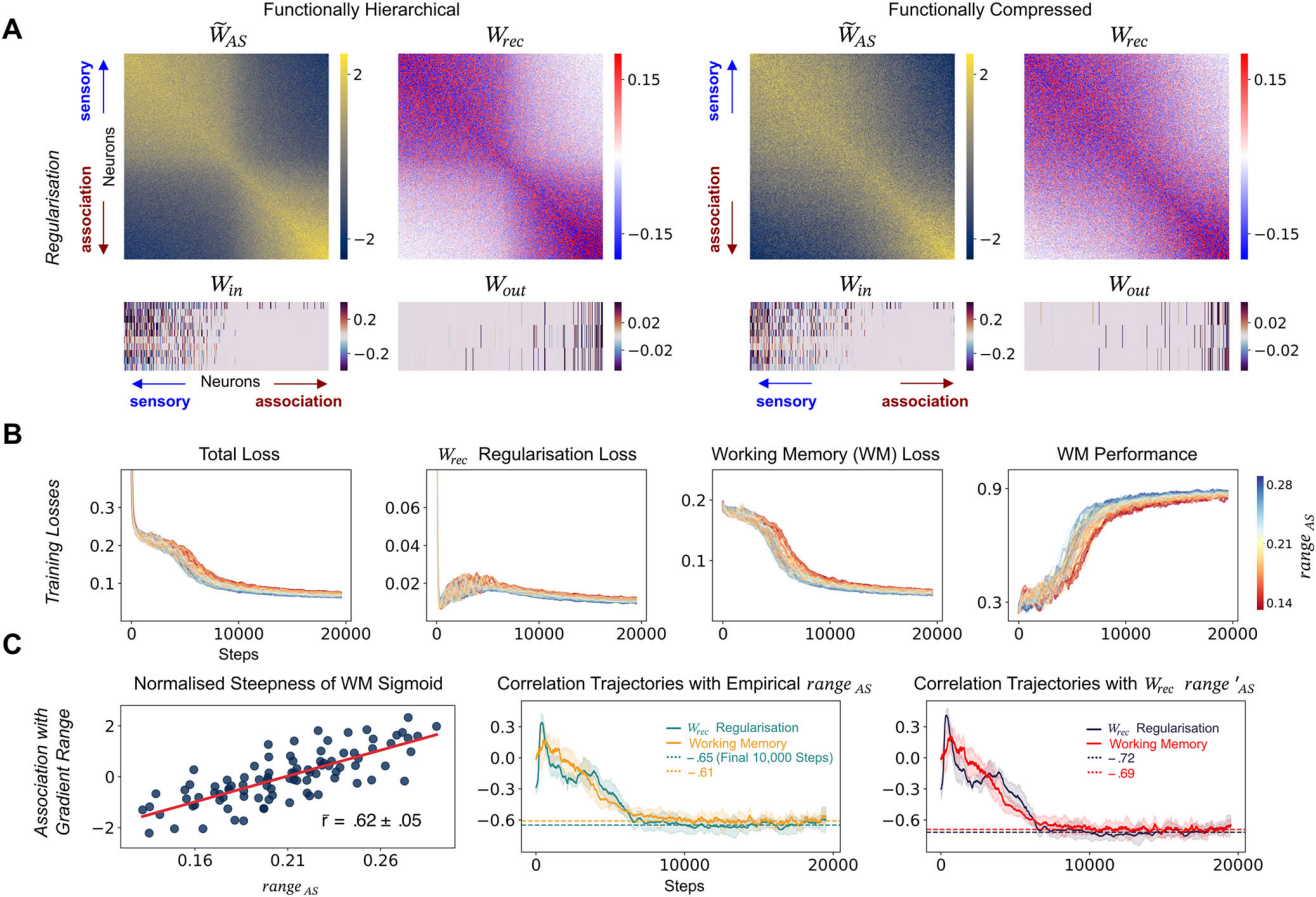
Learning dynamics of association-sensory gradient (*ψ*_AS_)—regularised recurrent neural networks. **A** Example *z*-scored AS matrices *W*_AS_ derived from *ψ*_AS_ (see Figs. 2 and 4), shown for functionally hierarchical (left) and functionally compressed (right) networks, along with the corresponding regularised recurrent (*W*_rec_), input (*W*_in_), and output (*W*_out_) weight matrices post-training. The *W*_rec_ regularisation enforces the AS organisational pattern in *W*_AS_, while the *W*_in_ and *W*_out_ regularisations facilitate input loading and prediction routing based on nodal specialisations implied by *ψ*_AS_ (“Methods” subsection “Recurrent neural network, working memory tasks, and regularisations”). **B** Training curves for total loss, *W*_rec_ regularisation loss, working memory (WM) task loss, and WM performance, each smoothed over 500 steps. Colour code reflects the *ψ*_AS_ used to generate *W*_AS_, with bluer lines corresponding to broader AS gradient ranges. **C** Correlation between the empirical range_AS_ used for ${W}_{{{\rm{AS}}}}$ generation and the steepness parameter $k$ of sigmoid fit to the WM performance, $f\left(x\right)=L/(1+{e}^{-k(x-{x}_{0})})$ (left), correlation trajectories between range_AS_ and both *W*_rec_ regularisation loss (green) and working memory loss (yellow; middle), as well as the trajectories using range_AS_’ computed from trained *W*_rec_ (right).

Regularisation had three layers (Fig. 6A):

1. Recurrent connectivity constraint—a mean-squared error loss pulling the *z*-scored absolute recurrent connectivity *|W*_rec_ *|* toward the *z*-scored *W*_AS_, preserving sign flexibility while enforcing AS geometry;
2. Input loading—an L1 term directs lower-order (sensory) units to receive external inputs; and
3. Output routing—a complementary L1 term encourages higher-order (associative) units to drive decisions.

To reduce computational load, our RNN training was based on a single site (Hartford, selected for the largest balanced cohorts; *N*_HC_ = 45, *N*_SZ_ = 47). We trained with five independent random initialisations (seeds) per subject (92 × 5 networks) for 20,000 steps, smoothed learning curves with a 500-step moving average to reveal denoised trends, and tracked Spearman correlations between range_AS_ and both task loss and weight-regularisation loss throughout training.

During the initial phase, total losses did not exhibit marked divergence across networks with varying ${{{\rm{range}}}}_{{{\rm{AS}}}}$. However, after ~4000 steps, those constrained by a broader AS gradient descended more steeply, converging onto lower plateaus (see colour-coded curves in Fig. 6B). Meanwhile, *W*_rec_ regularisation loss *L*_weights_ initially reached a minimum, followed by an oscillatory phase, diverging and stabilising according to range_AS_ as WM learning intensifies. Correspondingly, WM performance displayed an emergent separation by range_AS_, with broader eigenmodes yielding steeper performance gains. Sigmoid fits to the WM trajectories showed a positive correlation between steepness and range_AS_ (Fig. 6C; mean Spearman’s *r* = 0.62 ± 0.05 across seeds). The WM trajectories of task variants were similar and plateaued at comparable levels (Supplementary Fig. 2). Overall, this pattern suggests a two-stage learning process—networks first aligned with the AS gradient constraints, then adaptively reconciled that alignment to meet task demands.

Networks endowed with a broader gradient more readily accommodated both regularisation and functional demands, more efficiently adopting AS-like structure while simultaneously advancing toward stronger WM performance. This was evidenced by systematic decays in the correlation between range_AS_ with both WM (*L*_task_) and regularisation (*L*_weights_) losses, which stabilised by ~10,000 steps at −0.61 ± 0.04 and −0.65 ± 0.06, respectively (Fig. 6C). Furthermore, similar correlation trajectories were observed using the gradient range computed on trained recurrent matrices *W*_rec_, with *L*_task_–range_AS_’ and *L*_weights_–range_AS_’ correlations settling at −0.69 ± 0.04 and −0.72 ± 0.05, respectively, reinforcing the WM task advantage conferred by broader gradients. RNN learning and gradient range associations remained consistent across the tested *W*_AS_ hyperparameter grid ($\gamma \in \{\mathrm{0.10,0.20,0.30}\}$, $\sigma \in \{\mathrm{0.02,0.05,0.07}\}$; Supplementary Fig. 3).

### Stability during delay epochs via linearisation around fixed points

**Fig. 7:**
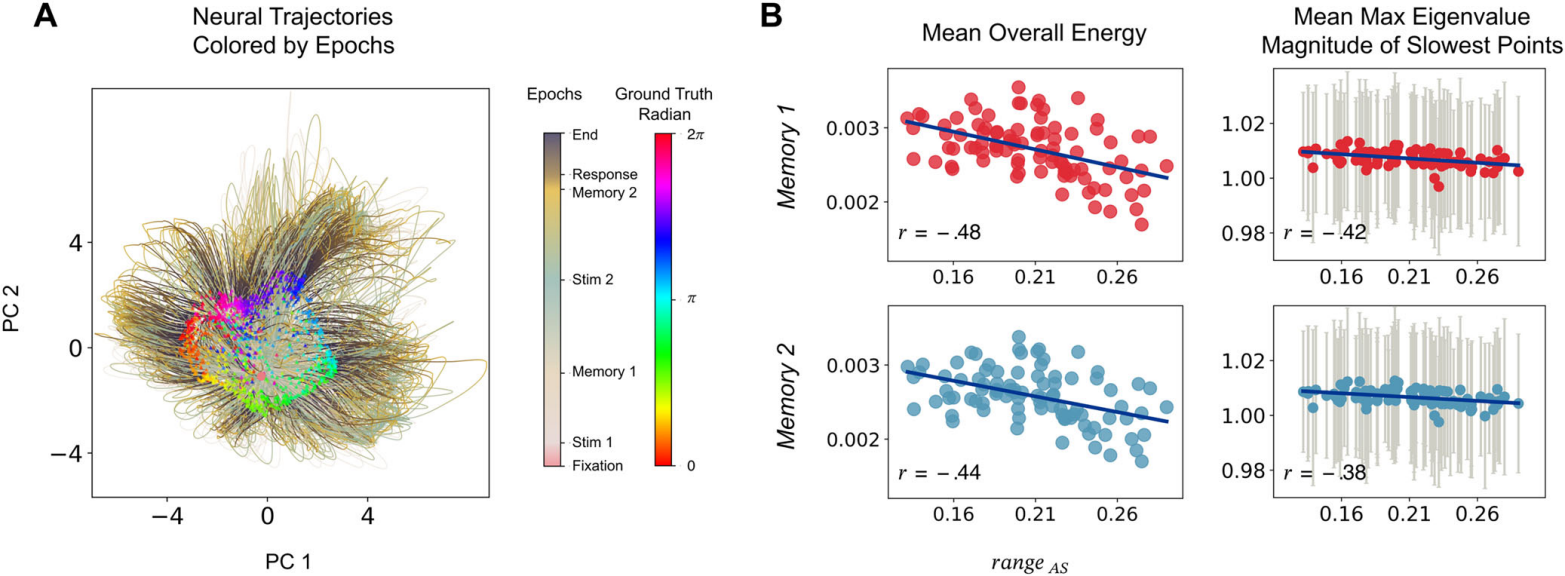
Greater association-sensory gradient (*ψ*_AS_) range is associated with more stable network states during memory delay. **A** Low-dimensional neural trajectories for a trained RNN performing Delayed Decision-Making (Dly DM) 1 task. Each trajectory (of a trial) is colour-coded by epoch, and trajectory endpoints are colour-coded by the target stimulus radian. Neural states for slow point optimisation were sampled from Memory epochs. **B** Mean energy of all slow points and mean maximum |λ| of the filtered slow points (with error bars denoting the standard deviations) both showed negative correlations with the range of *ψ*_AS_ used to regularise the RNN.

After showing that a broader *ψ*_AS_ accelerates and deepens WM task learning, we sought deeper mechanistic insight by assessing the stability of the network’s mnemonic states. We therefore performed fixed point (more precisely, slow points) analysis during the two post-stimulus delay epochs (Memory 1 and Memory 2; Fig. 7A), where the network settles into population states that maintain stimulus information once input ceases. Candidate states *h* were sampled from each trained RNN optimised toward minimal update ${||h}-F(h,u){||}$; those with small residual motion represent locations where network activity effectively stabilises under constant input *u* (see “Methods” subsection “Fixed point linearisation analysis”)^33,40^.

To focus on functionally relevant slow points, we ranked the ~1000 candidates per epoch by this residual motion, i.e., the energy, and retained the 100 lowest-energy points (≈top 10%). Lower energy denotes the state is closer to an exact fixed point; the filtering, hence, sharpens stability estimates. For each retained point, we computed the Jacobian $J({h}^{* })$; its largest eigenvalue magnitude |*λ*| indexes the most unstable direction. Averaging these maxima served as a network-level stability metric.

Across all task rules and seeds, the average energy cutoffs (for filtering) were 1.54 × 10^−6^ for Memory1 and 1.33 × 10^−6^ for Memory 2, with lower cutoffs seen in networks with higher range_AS_ (Memory 1: Spearman’s *r* = −0.26, *p* = 0.02; Memory 2: *r* = −0.23, *p* = 0.03). Correspondingly, the mean energy of all slow points showed significant negative correlation with range_AS_ (Memory 1: *r* = −0.48, *p* < 0.001; Memory 2: *r* = −0.44, *p* < 0.001; Fig. 7B), as well as with range_AS_’ of trained RNNs (*W*_rec_; Memory 1: *r* = −0.50, *p* < 0.001; Memory 2: *r* = −0.45, *p* < 0.001). These observations suggest that systems with broader gradient dispersion converge to lower-energy slow points, with their neural states functionally closer to true fixed points.

Furthermore, analysis of local stability via the maximum eigenvalue magnitudes revealed that networks with higher ${{{\rm{range}}}}_{{{\rm{AS}}}}$ showed smaller mean maximum |*λ*| at both memory epochs (Memory 1: Spearman’s *r* = −0.42, *p* < 0.001; Memory 2: *r* = −0.38, *p* < 0.001; Fig. 7B), and similarly for range_AS_’ (Memory 1: *r* = −0.42, *p* < 0.001; Memory 2: *r* = −0.37, *p* < 0.001). Recall that in the unstable regime |*λ*| > 1, approaching unity corresponds to dynamics along the corresponding eigenvector at that state, which are closer to marginal stability. These results indicate that even in the most unstable directions, networks with a broader gradient range achieve greater local asymptotic stability of memory dynamics. Taken together, our findings establish a link between a greater association-sensory eigenmode range and more dynamically stable maintenance of states during memory delay.

## Discussion

In this study, we focused on the most overarching organisational axis of functional brain systems, the association-sensory (AS) gradient. Rather than discrete functional modules, the AS gradient encodes a continuous organisation of connectivity patterns from unimodal to transmodal regions. Our experimental analyses revealed that schizophrenia is characterised by a de-differentiation of the AS gradient and a dampened relationship between gradient extremes and neural timescales, with modest but reliable effect sizes, consistent with a contraction of the brain’s hierarchical organisation in both spatial and temporal domains. Meanwhile, in our theoretical branch, we show that more diffuse and well-differentiated AS systems support more adaptive learning of a canonical working memory (WM) computation and, critically, maintain network states more stably during memory delays.

The compression of the principal AS gradient in schizophrenia aligns with prior evidence, indicating a reduced differentiation (i.e., less differentiated specialised systems) along the brain’s most global organisational dimension^14,15,47^. Discrete dysconnectivity studies corroborate this pattern, showing diminished within-network coherence in both sensory and higher-order systems, as well as less efficient multistep connectivity propagation along the AS hierarchy in schizophrenia^14,48–50^. As shown descriptively, broader *ψ*_AS_ range reflects a more pronounced hierarchical arrangement, with steeper connectivity drop-off along *ψ*_AS_ and stronger local connectivity among similarly specialised nodes, and sparser connectivity among distant gradient extremes. In contrast, a compressed gradient corresponds to an attenuated local-distant contrast, resulting in flatter, less differentiated architecture that diverges from the sparsely interconnected specialised compositions believed to foster adaptive, concurrent, and locally segregated processes^51,52^. Correspondingly, gradient range showed a positive association in the whole sample, and was negatively associated with positive symptoms in the schizophrenia cohort, both with small effects. Beyond connectivity, the AS organisation is evident in latent dimensions of neural timeseries, in dynamical property decomposition, and in the formation of low-energy attractor states that exert a “gravitational pull” on brain activity configurations^53–55^. This reduced specialisation in connectivity space may compromise stability of neural dynamics and computations. Accordingly, we first characterised how neural timescale varies along *ψ*_AS_ empirically; we then assessed dynamical stability using *ψ*_AS_-regularised RNNs and fixed-point analysis.

Our analyses revealed that neural timescales tend to lengthen with greater functional specialisation. Dynamic profiles of the brain are not merely local phenomena; for instance, regions with stronger power in lower frequencies, akin to low-pass filtering, and slower timescales display higher functional connectivity (FC), both globally and within unimodal and transmodal systems^17,56^. In parallel, regional dynamic profiles are more similar within rather than between functional modules, and longer timescale has been specifically linked to within-community integration, where hubs at the core of specialised systems can facilitate segregated information processing^25^.

In contrast, faster, more flexible dynamics, indicative of enhanced sensitivity toward instantaneous signals, may characterise connector hubs that bridge distinct functional subsystems, as suggested by their shifting synchronisation and modular allegiance over time^57^. These hubs dynamically coordinate inter-module communication without overloading each specialised system, thereby preserving local autonomy^58^. Functionally, such intermediate systems may act as saddle points, routing transient signals onto the slower, more specialised regions that then serve as stable attractors for sustained encoding^40^.

We did not explicitly model the unimodal-transmodal dichotomy, where prior work has reported overall faster dynamics in unimodal systems hosting perceptual processes and slower transmodal dynamics supporting integrative functions^17,21,22^. Instead, our analysis offers a complementary perspective on spatial-temporal convergence, where specialised anchors within each subsystem display slower neural dynamics. Nevertheless, although the quadratic model outperformed a simple linear fit, the mapping remains individually heterogeneous (e.g., coefficient strength) and thus requires further validation. Crucially, this *ψ*_AS_ -timescale relationship is an empirical mapping and does not establish a mechanistic account linking gradient geometry to autocorrelation decay.

The AS gradient-timescale mapping in schizophrenia showed a less pronounced hierarchical organisation of neural dynamics, with diminished relative slowness at the gradient extremes. Previous findings suggested globally shortened timescales, symptom-specific hierarchical disruptions, and instability in dynamic FC that indicate more rapidly fluctuating neural synchronisations in schizophrenia^23,28,59,60^. The observed reduction in slowness at specialised poles may further reflect destabilised cortical attractor states that are prone to noise-provoked random transitions, undermining sustained information encoding^61,62^. Having linked spatial gradients to temporal dynamics empirically, we next interpret how these observations resonate with our RNN results.

In our *ψ*_*AS*_-regularised RNNs, networks with a higher gradient range achieved more efficient learning and lower task loss, while maintaining lower regularisation loss against the AS connectivity constraint. Such well-differentiated functional systems may be less susceptible to computational interference. This synergy between the high-range regularisation and the task demand is consistent with the simultaneous yet autonomous computations performed by specialised neural systems, coordinated via connector nodes in the brain^58,63,64^.

Using our *W*_*AS*_ generative model, we integrated stereotypical features of brain network topology—functional modularity, sparsity between specialised systems, small-worldness—yet sustained a continuous approach without imposing rigid modules. These topological properties are theorised to arise from the joint optimisations of metabolic costs (i.e., network development and maintenance) and information-processing demands^65,66^. Theoretical work suggests that balancing local cohesion and global diffusion fosters effective signal spreading and computations^67^. On the other hand, functional demands actively sculpt network organisation, as evidenced by RNN hidden units that self-organise into compositional, specialised functional clusters, thereby supporting cognitive flexibility^32^. Similarly, explicitly enforcing biophysical wiring constraints in RNNs can generate structural motifs and functional clustering reminiscent of brain networks when optimised for inference tasks^66^. By incorporating a gradient-constrained connectivity, our framework echoes with these findings, suggesting that a more diffuse functional organisation, featuring steeper local-distant contrast, may achieve an economical balance between computational efficiency and connectivity cost, a principle seemingly shared between biological brains and artificial RNNs.

Moreover, greater *ψ*_AS_ range was associated with more stable, slowly evolving neural states during memory delay periods. This corroborates studies showing that artificial networks with modular, hierarchical functional communities exhibit persistent activities and stable, scalable activation spread, unlike random networks^68–70^. In the brain, stable maintenance of whole-brain activity patterns correlates with greater WM performances, whereas in schizophrenia, brain state stability is reduced, harder to control, and more vulnerable to perturbations^71–73^. This pattern aligns with dynamical system models of WM deficits in schizophrenia, which posit a flattened attractor landscape and corresponding unstable, lower-capacity memories susceptible to distractibility^26,74–76^. Our RNN simulations align with this view and, critically, offer a testable hypothesis, where task-based designs that jointly model gradient geometry, brain state stability, and trial-wise task performances can determine whether AS contraction is associated with reduced state maintenance and cognition. Taken together, we present two complementary perspectives on the instability of neural dynamics in schizophrenia; one describing empirical timescale flattening along *ψ*_AS_, and another providing a theoretical demonstration of more unstable fixed point dynamics exhibited by low-range RNNs.

Importantly, the relationship between *ψ*_AS_ range and RNN learning dynamics should be interpreted as dependent on the computational regime being studied. The delayed decision paradigm requires preservation of information across time, hence favouring networks that are able to form stable internal states. Multitask RNN studies show that different task categories rely on distinct dynamical motifs, including attractors, decision boundaries, rotations, and that computation is reconfigured to context by reusing and recombining these motifs^32,33^. Therefore, a given structural bias could result in regime-specific effects. Accordingly, while a broader *ψ*_AS_ range benefited the learning and stability in computational regimes requiring state maintenance under our theoretical framework, its interaction across different regimes (e.g., rapid stimulus-response) could be investigated in future work, both computationally and empirically.

All FC used for gradient extraction and computational models was derived from resting-state data. By imposing these FC-based constraints, we capitalise on a direct proxy for interareal communication, as the activity flow principle—modelling a region’s activation as the sum of inputs weighted by empirical resting-state FC—predicted task-state activations^54,77,78^. Critically, the brain’s resting-state network architecture fundamentally shapes its task-based organisation and maintains a full repertoire of interacting networks even at “rest” ^58,79,80^. As a regulariser, the resting architecture also highly aligns with multitask FC, reinforcing the notion of a stable functional scaffold at rest^81^. Although task-fMRI typically reveals necessary network integration when tasks are not fully automated, training has been shown to drive more pronounced functional segregation of specialised systems, thereby boosting execution autonomy and neural efficiency^82^^,^^83^.

In this context, our study focuses on the schizophrenia-implicated AS gradient, examining its role within this standard functional architecture and its computational consequences. Notably, task-state AS gradient range has been shown to be predictive of cognition, motivating further validation of our observations with task-based gradients in schizophrenia and optimisation of gradient-based regularisation strategies^84^. While our current RNNs employ regularisation based solely on the AS gradient, future studies should consider incorporating additional gradients, such as the cross-sensory gradient, to explore how multiple connectivity dimensions interact to shape computations^83,84^.

To conclude, through a continuous-network perspective, we investigated dysconnectivity in schizophrenia along the brain’s principal AS gradient and its dynamical and computational consequences. Empirically, we showed that schizophrenia displays gradient compression indicative of reduced hierarchical functional specialisation, paralleled by diminished temporal differentiation. Theoretically, embedding these empirical gradient structures into RNNs suggested that AS system compression can destabilise fixed point dynamics critical for noise-robust computational stability. This integrated experimental-theoretical framework not only advances connectivity-to-computation insights into schizophrenia, but also establishes a foundational approach for future neuroAI research. In particular, it sets a precedent for designing artificial systems informed by spectral properties of brain connectivity, enabling wider empirically-grounded theoretical explorations of neural computation.

## Methods

### Functional MRI

The resting-state fMRI data from the BSNIP consortium consisted of 186 healthy controls and 169 schizophrenia patients; identical diagnostic and recruitment approaches were applied across sites, and all subjects underwent a 5-min resting-state scan on a 3-T scanner^41,42^. The fMRI images were preprocessed by a prior pipeline that included slice-time correction, rigid-body head motion correction, co-registration to the T1-weighted anatomic volume, transformation to MNI152 standard space, wavelet despiking of motion artefacts, regression of 12 motion parameters, and spatial smoothing at 6 mm FWHM^85,86^. Cortical parcellation was conducted using the Glasser atlas with 360 regions^87^. Time series were then bandpass filtered using wavelet scales 2 and 3 (covering 0.028–0.167 Hz across the dataset), leveraging wavelets’ simultaneous time-frequency localisation to mitigate the influence of long-memory processes^88–92^. Functional connectivity (FC) matrices were derived as the pairwise Pearson correlation between time series from all region pairs.

### Spatial gradients via diffusion map

Pairwise affinity $A$ from each sparsified FC matrix was computed using the normalised angle kernel, defined as $A\left(i,j\right)=1-\arccos (\frac{x{y}^{T}}{{||x||||y||}})/\pi$, with x and y denoting the connectivity arrays of nodes $i$ and $j$. This transforms cosine similarity into a non-negative affinity bounded in $[\mathrm{0,1}]$. To extract gradients, we first constructed a kernel $K={D}^{-\alpha }A{D}^{-\alpha }$, where *D* is the degree matrix ${D}_{{ii}}={\sum }_{j}{A}_{{ij}}$. We set $\alpha =0.5$, corresponding to Fokker-Planck diffusion, a normalisation step that reduces sensitivity to sampling density while preserving global structure in the embedded space^93^. A Markov diffusion operator was then computed as $P={D}_{K}^{-1}K$, where ${({D}_{K})}_{{ii}}={\sum }_{j}{K}_{{ij}}$, ensuring each row sums to 1 so that $P$ defines a transition matrix of one-step probabilities. As setting a single diffusion time $t$ confines the embeddings $\{{\lambda }_{k}^{t}{\psi }_{k}\}$ to a particular scale, the eigenvectors of $P$ were weighted using multiscale eigenvalue aggregates (i.e., ${\sum }_{t=1}^{\infty }{\lambda }_{k}^{t}={\lambda }_{k}/(1-{\lambda }_{k})$). This incorporates the diffusion process across multiple time scales to produce the final set of scaled diffusion coordinates defining functional gradients^93–95^. This process was implemented using the BrainSpace toolbox^93^.

A quantitative measure of network hierarchy encoded by each gradient is given by its range, ${{\rm{range}}}\left({\psi }_{k}\right)=\max \left({\psi }_{k}\right)-\min \left({\psi }_{k}\right)$, where a larger range indicates stronger differentiation among network elements along that diffusion axis. Individual embeddings were aligned to the group-level representation using orthogonal Procrustes transformations, which preserve inter-point Euclidean relations through rigid rotations without scaling, ensuring dimensional consistency across all embeddings.

Connectivity matrices were sparsified at three density thresholds, retaining the top 40, 50, and 60 edges per node (approx. 11%, 14%, and 17% of the strongest connections, respectively). These thresholds align with thresholds used in previous studies and serve to balance the signal-to-noise ratio. After confirming the robustness of gradient patterns across all thresholds, we adopted the intermediate density of 50 edges per node (14%) for subsequent analyses.

### Neural timescale estimation

We define the timescale $\tau$ of a neural time series {*X*_*t*_ } by the exponential decay rate of its autocorrelation function (ACF), ${{\rm{ACF}}}\left(s\right){\mathbb{=}}{\mathbb{E}}\left[{X}_{t}{X}_{t+s}\right]{\mathbb{/}}{\mathbb{E}}\left[{X}_{t}^{2}\right]$. A typical estimation approach is to fit an exponential directly to empirical ACF values. However, for finite sample sizes, such estimates can exhibit systematic biases that depend on both length of the observed series and the underlying ACF, see Zeraati et al.^45^.

To mitigate these biases, we model {*X*_*t*_ } as an Ornstein-Uhlenbeck (OU) process, a Gauss-Markov process with mean reversion and a closed-form ACF. It satisfies the stochastic differential equation

3 $$d{X}_{t}=-\frac{1}{\tau }{X}_{t}{dt}+\sigma d{W}_{t},$$

where the deterministic term $-\frac{1}{\tau }{X}_{t}{dt}$ (with characteristic timescale *τ* > 0) pulls *X*_*t*_ toward its long-run mean (assumed zero in mean-centred data), while the stochastic term $\sigma d{W}_{t}$ (with noise amplitude *σ* > 0 and *W*_*t*_ as a standard Wiener process) injects noise. In stationarity, its autocorrelation decays exponentially as:

4 $${ACF}\left(s\right)={e}^{-\frac{\left|s\right|}{\tau }}$$

Discretising at sampling interval ∆*t* yields a Markov sequence where

5 $${\mathbb{E}}\left[{X}_{t+1}\left|{X}_{t}\right.\right]={X}_{t}{e}^{-\frac{\Delta t}{\tau }},$$

6 $${Var}\left[{X}_{t+1}\left|{X}_{t}\right.\right]=D\tau (1-{e}^{-\frac{2\Delta t}{\tau }}),$$

with $D={\sigma }^{2}/2$. By Markov property, the joint likelihood of the full observed sequence is the product of conditional Gaussian densities

7 $$L\left(\tau ,D\right|{X}_{0},{X}_{1},\ldots ,{X}_{T})=\mathop{\prod }\limits_{t=0}^{T-1}\frac{1}{\sqrt{2\pi {Var}\left[{X}_{t+1}\left|{X}_{t}\right.\right]}}\exp \left[-\frac{{({X}_{t+1}{\mathbb{-}}{\mathbb{E}}\left[{X}_{t+1}\left|{X}_{t}\right.\right])}^{2}}{2{Var}\left[{X}_{t+1}\left|{X}_{t}\right.\right]}\right]$$

Taking the negative log-likelihood (NLL) in terms of parameters $\tau ,D$ gives

8 $$-\log L\left(\tau ,D\right)=\mathop{\sum }\limits_{t=0}^{T-1}\left[\frac{1}{2}\log \left(2\pi D\tau \left(1-{e}^{-\frac{2\Delta t}{\tau }}\right)\right)+\frac{{\left({X}_{t+1}-{X}_{t}{e}^{-\frac{\Delta t}{\tau }}\right)}^{2}}{2D\tau \left(1-{e}^{-\frac{2\triangle t}{\tau }}\right)}\right]$$

Minimising this NLL with respect to *τ* and *D* produces maximum likelihood estimates (MLE) $\hat{\tau }$ and $\hat{D}$.

For comparison, a computationally simpler alternative is to compute the empirical autocorrelations $\hat{\rho }\left({\ell}\right)$ successive lag ${\ell}$ up to a predefined maximum, then fit the resulting values jointly to an exponential decay ${e}^{{-}{\ell}{\Delta}t/\tau }$ with least-squares. However, each $\hat{\rho }\left({\ell}\right)$ carries estimation variability, in contrast to OU-MLE inferring $\tau$ from a complete probabilistic model of the data. The Gaussian assumption underlying the OU was empirically validated in a random sample of subjects by testing for stationarity of the preprocessed time series (Augmented Dickey-Fuller) and for Gaussianity of both the OU-fitted residuals and time series (Shapiro–Wilk) in randomly sampled subjects. Having established that OU-MLE provides reliable timescale estimates, we then examined how intrinsic timescales align with the spatial gradient.

### Spatial-temporal convergence

To assess the relationship between the timescale $\tau$ and association-sensory (AS) gradient *ψ*_AS_, both quantities were normalised within each subject using min-max scaling, ${{\rm{MinMaxScalar}}}\left(x\right)=\frac{x-\min x}{\max x-\min x}$, to account for cross-subject variability in absolute ranges and to ensure comparability of relative patterns. We then compared a linear $\hat{\tau }$ $\approx {\beta }_{1}{\hat{\psi }}_{{{\rm{AS}}}}+{\beta }_{0}$ and quadratic $\hat{\tau }\approx {\beta }_{2}{\hat{\psi }}_{{{\rm{AS}}}}^{2}+{\beta }_{1}{\hat{\psi }}_{{{\rm{AS}}}}+{\beta }_{0}$ model, where the quadratic term *β*_*2*_ captures potential curvature indicative of hierarchical temporal organisation along the gradient. Model selection was guided by paired AIC comparison across subjects. For the selected model, the coefficient of interest—i.e., *β*_*1*_ for linear, *β*_*2*_ in quadratic—was compared between cases and controls using multiple regression with adjustment for relevant confounds.

### Generative model of association-sensory matrix ${W}_{{{\rm{AS}}}}$

For the theoretical analysis, to investigate how variations in AS gradient *ψ*_AS_ influence working memory computations with recurrent neural networks (RNNs), we developed a generative model to construct subject-specific AS connectivity matrices *W*_AS_ using their own empirical *ψ*_AS_, while holding the model parameters fixed. This model is designed to convert *ψ*_AS_ into a recurrent-weight constraint whose principal gradient *ψ*_AS_’ preserves each subject’s nodal hierarchy and gradient range. These matrices were then used to regularise RNNs, enabling us to directly assess the effect of gradient range (AS differentiation) on task learning and dynamical stability.

Our generative process combines distance-dependent connectivity decay with hierarchical scaling, building on empirical observations from Results: Association-sensory gradient range and network specialisation. For each subject, we first modelled the observed connectivity decay with gradient separation by defining a locality matrix

9 $${W}_{L}=\alpha D+\beta$$

10 $${D}_{{ij}}=\left|{\psi }_{{{\rm{AS}}}}\left(i\right)-{\psi }_{{{\rm{AS}}}}\left(j\right)\right|$$

Here, *D*_*ij*_ is a one-dimensional distance along the subject’s *ψ*_*AS*_ axis, and because *α* < 0, *W*_*L*_ decreases monotonically with increasing AS-gradient separation. We fit linear decay models at each node on the mean connectivity matrix ${\bar{W}}_{{{\rm{FC}}}}$ across all subjects, then averaged those fits across nodes to obtain the node-invariant parameters *α* = −1.35 and *β* = 0.40. Although *W*_*L*_ encodes local granularity along each subject’s *ψ*_AS_, it does not emphasise the empirically observed steeper local-distant contrast at the sensory and association extremes. To incorporate this effect, we elementwise multiplied *W*_*L*_ by a hierarchical-scaling term

11 $${W}_{G}=\widetilde{\psi }{\widetilde{\psi }}^{T}+1,$$

12 $$\widetilde{\psi }=\frac{{\psi }_{{AS}}}{\gamma }.$$

This positive modulation augments within-pole coupling and attenuates cross-pole coupling, since ${{W}_{G}}_{{ij}} > 1$ when *ψ*_*i*_ and *ψ*_*j*_ share the same sign and ${{W}_{G}}_{{ij}} < 1$ when they have opposite signs. This modulation is strongest for nodes with larger |*ψ*_*AS*_ |, effectively steepening the local-distant contrast at gradient extremes, aligning with the empirical pole-centred geometry. The scaling parameter *γ* was chosen based on gradient recovery, with optimal recovery observed within 0.15–0.25. We selected an intermediate *γ* = 0.20 achieving realistic connectivity ranges (average min = 0.08, max = 0.57, mean = 0.32) while ensuring gradient fidelity. Using the same evaluation pipeline, the combined ${W}_{L}\odot {W}_{G}$ outperformed simpler distance-only and outer-product constructions in preserving both within-subject *ψ*_AS_ ordering and cross-subject gradient range.

However, this deterministic mapping ${W}_{L}\odot {W}_{G}$ can interact with the fixed-density threshold used for empirical gradients to produce a discretised similarity structure, including an inflated intermediate grouping between poles, when we re-extract gradients from the generated matrix (see “Results” subsection “Generative model of association-sensory weight matrix from *ψ*_AS_ embeddings”; Fig. 5B). We therefore introduced low-intensity Gaussian noise with standard deviation *σ* = 0.05 to reduce these sharp threshold-induced artefacts while preserving the underlying hierarchy. We selected $\sigma$ by incremental testing from 0 to 0.10 in 0.01 steps to ensure robust recovery of gradient range (boosting correlation from 0.76 to 0.94) without distorting the overall hierarchy.

Our final generative model was thus:

13 $${W}_{\!{{\rm{AS}}}}={W}_{L}\odot {W}_{G}+{\mathscr{N}}\left(0,{\sigma }^{2}\right)$$

To evaluate the robustness of downstream RNN conclusions to these generative hyperparameters, we trained RNNs across a plausible parameter grid with $\gamma \in \{\mathrm{0.10,0.20,0.30}\}$ and $\sigma \in \{\mathrm{0.02,0.05,0.07}\}$, and report the resulting sensitivity analysis in Supplementary Note 3 and Supplementary Fig. 3.

### Recurrent neural network, working memory tasks, and regularisations

We examined Euler-discretised continuous-time RNNs^32,33^, following leaky integration update:

14 $${h}_{t+1}=\left(1-\alpha \right){h}_{t}+\alpha f({W}_{{{\rm{rec}}}}{h}_{t}+{W}_{{{\rm{in}}}}{u}_{t}+b+{\xi }_{t})$$

15 $$f\left(x\right)=\max (0,x)$$

Here, the integration constant $\alpha =\Delta t/\tau$ governs information retention and was set to 0.20. *W*_in_ and *W*_rec_ are the input and recurrent weight matrices of dimensions *N*_in_ × *N*_neuron_ and *N*_neuron_ × *N*_neuron_, loading external inputs *u*_*t*_ and upstream activities to downstream units. The noise term *ξ*_*t*_ comprises *N*_neuron_ independent Gaussian white noise processes scaled by $0.05\sqrt{2{\alpha }^{-1}}\approx 0.158$. Output units *z* were computed via linear readout

16 $${z}_{t}={W}_{{{\rm{out}}}}{h}_{t}+{b}_{{{\rm{out}}}}$$

with ${W}_{{{\rm{out}}}}\in {{\mathbb{R}}}^{{N}_{{{\rm{neuron}}}}\times {N}_{{{\rm{out}}}}}$ and bias *b*_out_.

Working memory tasks were adopted from delay decision making (Dly DM) task family (five variants; Fig. 4) in Yang et al.^32^ and Driscoll et al.^33^. Briefly, noisy inputs *u* included a one-dimensional fixation signal, four-dimensional stimulus channels, and five-dimensional rule one-hot vectors. Stimuli were angles *θ*$\in$ [0,2*π*), presented in two modalities as [*A*sin*θ, A*cos*θ*] pairs, with *A* modulating strength. Trial epochs comprised initial fixation, stimulus 1, memory 1, stimulus 2, memory 2, and response, with each duration uniformly sampled from preset ranges. In “Dly DM 1 & 2” tasks, only one modality was available; in “Ctx Dly DM 1 & 2” tasks, both modalities appeared, but only one was attended; and in “Mult Dly DM” tasks, both modalities informed the decision. The task objective was to accurately select the stimulus direction of highest intensity, with performance deemed accurate if the network’s final response angle *ϕ* lay within ±*π*/5 of the target. Target output $\hat{z}$ was a fixation component plus [sin*ϕ,* cos*ϕ*] encoding the chosen angle. We computed squared error loss between $\hat{z}$ and network output, weighted cost mask accentuating post-response errors. Training was conducted simultaneously on all five task variants over 20,000 iterations using minibatches of 128 trials, with each unique batch randomly drawn from one of the variants with equal probability. Networks were optimised with Adam (learning rate = 10^−3^), a variant of stochastic gradient descent, to minimise the total loss (see below)^96^.

To regularise *W*_rec_—initialised with random orthogonal initialisation—toward hierarchical patterns resembling the AS constraint *W*_AS_ generated from *ψ*_AS_, while minimising sensitivity to raw magnitude, we defined a weight-regularisation loss *L*_weights_ as the mean-squared error between *z*-scored ${\widetilde{W}}_{{{\rm{AS}}}}$ and *z*-scored, absolute ${\widetilde{W}}_{{{\rm{rec}}}}$

17 $${{\ell}}_{{{\rm{weights}}}}={{\rm{MSE}}}\left({\widetilde{W}}_{{{\rm{AS}}}},{\widetilde{W}}_{{{\rm{rec}}}}\right)$$

Both matrices were mean-centred and scaled to unit variance, ensuring organisational alignment rests on connectivity pattern rather than penalising overall weight scales. Taking |*W*_rec_| before standardisation aligns its relative magnitude profile with the functional interactions encoded in *W*_AS_, without constraining recurrent links to be excitatory or inhibitory, preserving functional flexibility.

To conform each network’s input and output structure with its intrinsic nodal AS specialisations, we imposed L1 penalties on *W*_in_ and *W*_out_. Specifically, *ψ*_AS_ was min-max scaled into ${\widetilde{\psi }}_{{{\rm{AS}}}}\in [\mathrm{0,1}]$, mapping sensory neurons toward 0 and association to 1. During training, we computed

18 $${{\ell}}_{{{\rm{in}}}}=\left\langle {\widetilde{\psi }}_{{{\rm{AS}}}}\odot \left|{W}_{{{\rm{in}}}}\right|\right\rangle ,$$

19 $${{\ell}}_{{{\rm{out}}}}=\left\langle ({1-\widetilde{\psi }}_{{{\rm{AS}}}})\odot \left|{W}_{{{\rm{out}}}}\right|\right\rangle$$

The network was therefore encouraged to direct inputs primarily into lower-order sensory units while routing predictions through higher-order association regions. Our total loss was defined as

20 $${{\ell}}_{{{\rm{total}}}}={{\ell}}_{{{\rm{task}}}}+{{\ell}}_{{{\rm{weights}}}}+{{\ell}}_{{{\rm{in}}}}+{{\ell}}_{{{\rm{out}}}}.$$

### Fixed point linearisation analysis

The dynamics of trained, *ψ*_AS_-regularised RNNs were examined through identifying fixed points—states h^*^ with which, as initial conditions, the system exhibits minimal motion and satisfies ${h}^{* }\approx F({h}^{* },u)$; *F* is the update rule, and *u* is an external input vector specifying the task condition^33,40^. Our fixed points included approximately fixed slow points where the system is not stationary but evolves minimally.

Near each slow point, h^*^ + *δh*_*t*_, the state update can be linearised through first-order Taylor expansion

21 $${h}_{t+1}=F\left({h}^{* }+\delta {h}_{t},u\right)\approx F\left({h}^{* },u\right)+J({h}^{* })\delta {h}_{t}$$

Where *J*(h^*^) is the Jacobian matrix evaluated at h^*^, with

22 $${J}_{{ij}}\left({h}^{* }\right)={\left.\frac{\partial {F}_{i}}{\partial {h}_{j}}\right|}_{h={h}^{* }}$$

The evolution of sufficiently small perturbations, *δh*_*t*_, around a slow point can thus be approximated as:

23 $$\delta {h}_{t+1}\approx J({h}^{* })\delta {h}_{t}$$

The local stability, i.e., whether the system converges to or diverges from h^*^, was assessed through the eigendecomposition of *J*(h^*^). The system contracts along a dimension (eigenvector) if the corresponding eigenvalue magnitude |*λ*| < 1 (locally stable), expands if |*λ*| > 1 (unstable), and is marginally stable if |*λ*| ≈ 1. Our analyses focused on post-stimulus delay epochs, where the network’s hidden activity holds stimulus information under the constant fixation input.

For each task variant and *ψ*_AS_-regularised RNN, we generated a batch of trials and sampled 1000 random neural states *h* from each of the Memory 1 and Memory 2 epochs. We then optimised these states by gradient descent on the energy function

24 $$q=\frac{1}{2}{\|h-F(h,u)\|}^{2},$$

to identify candidate slow points. From the resulting ~1000 slow points (per epoch), we sorted by energy, retained the 100 slowest-energy points, and computed at each the maximum eigenvalue magnitude |*λ*| of *J*(h^*^). Finally, we averaged (1) the energies of all slow points and (2) the |*λ*| maxima of the filtered subset across task variants and random seeds, then correlated both metrics with each network’s AS gradient range to assess how gradient dispersion relates to memory dynamics.

## Supplementary information

Supplementary_material_revision_feb26

## Data Availability

Data used in the preparation of this manuscript were obtained from the National Institute of Mental Health (NIMH) Data Archive (NDA; https://nda.nih.gov/). NDA is a collaborative informatics system created by the National Institutes of Health to provide a national resource to support and accelerate research in mental health. Dataset identifiers: NDAR ID: 2274. This manuscript reflects the views of the authors and may not reflect the opinions or views of the NIH or of the Submitters submitting original data to NDA.

## Code availability

All codes used in empirical and theoretical studies are available on the following GitHub repository: https://github.com/SubatA20/as_spec_rnn.
