# Supplementary material for: Association-sensory spatiotemporal hierarchy and functional gradient-regularised recurrent neural network with implications for schizophrenia
Source: NPJ Syst Biol Appl. 2026 Apr 30;12:100. doi: 10.1038/s41540-026-00727-x (PMC13338132; doi:10.1038/s41540-026-00727-x)
Supplement: Supplementary file 1 — Supplementary_material_revision_feb26 [file 41540_2026_727_MOESM1_ESM.pdf]

Supplementary material

Supplementary Note 1 Compressed association-sensory gradient in schizophrenia at different network density thresholds.

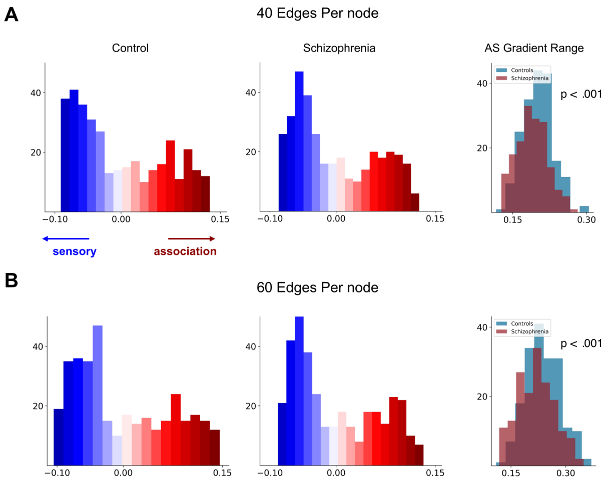

Supplementary Figure 1 The compression of AS gradient was consistently observed at sensitivity analyses under 40 (**A**) and 60 (**B**) edges per node.

Supplementary Note 2 Task performance across subtasks.

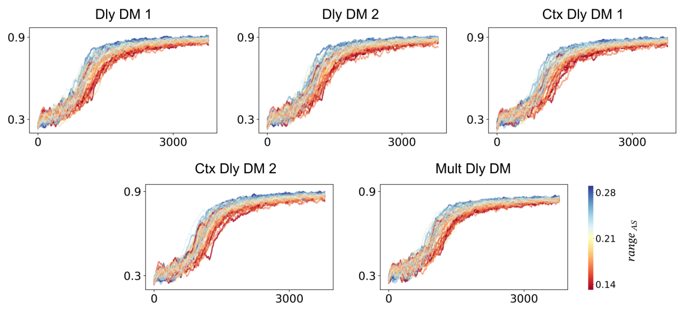

Supplementary Figure 2 Across all randomly alternating task variants, networks converged to comparable accuracies. Those regularised with higher association-sensory spectral range tended to rise more quickly and plateau higher.

Supplementary Note 3 Sensitivity analysis of RNN learning effects on W_{AS} generative model parameters γ and σ.

Our construction of the association-sensory constraint matrix W_{AS} combines a ψ_{AS}-distance-dependent connectivity decay with hierarchical scaling and a small stochastic term

$$W_{AS} = W_L \odot W_G + \mathcal{N}(0, \sigma^2),$$

$$W_L = \alpha D + \beta, \quad D_{ij} = |\psi_{AS}(i) - \psi_{AS}(j)|,$$

$$W_G = \tilde{\psi}\tilde{\psi}^T + 1, \quad \tilde{\psi} = \frac{\psi_{AS}}{\gamma}.$$

In the main analysis, we used $\gamma = 0.20$ and $\sigma = 0.05$ that supported construction of W_{AS} whose principal gradient aligns closely with the empirical ψ_{AS}.

To evaluate whether RNN learning depends on these specific choices, we repeated the RNN training on a subset of samples across a plausible grid of $\gamma \in \{0.10, 0.20, 0.30\}$ and $\sigma \in \{0.02, 0.05, 0.07\}$, where the resulting W_{AS} preserved robust gradient fidelity, with mean ψ_{AS}-ψ_{AS}' correlations of 0.96-0.98 and $range_{AS}$-$range_{AS}'$ correlations of 0.85-0.94. Due to computational constraints, we sampled a $N = 40$ subset from the 92 Hartford participants by stratifying subjects into quartiles of $range_{AS}$ and randomly sampling 10 each quartile with balanced controls and schizophrenia subjects.

For each γ-σ setting, the RNNs were trained with the same architecture and regularisation protocol aligned with the main analysis. We assessed the relationship between $range_{AS}$ and working memory (WM) loss (lower the better) as well as the steepness of WM performance trajectories (higher suggests faster performance gains) using Spearman correlation. Across tested parameter settings, the direction of effect was preserved, with $range_{AS}$ remaining negatively correlated with WM loss and positively correlated with learning steepness, and the correlation magnitude remained moderate to strong (Fig S3; Spearman's $r = -0.53$ to -0.69; 0.51 to 0.71). This shows the theoretical conclusions that broader ψ_{AS} range associates with faster learning and better converged WM performance under AS-regularised training are not driven by specific γ-σ setting

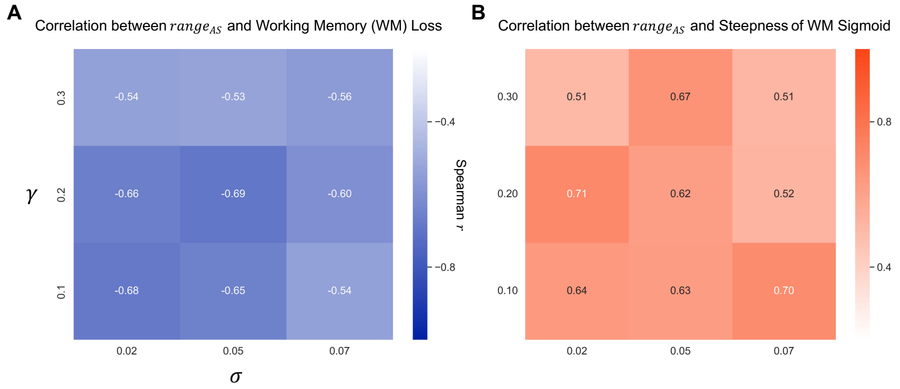

Supplementary Figure 3 Sensitivity of $range_{AS}$ and working memory (WM) task learning relationships to W_{AS} parameters γ and σ. (A) Spearman correlations between empirical $range_{AS}$ and final WM task loss across the grid of $\gamma \in \{0.10, 0.20, 0.30\}$ and $\sigma \in \{0.02, 0.05, 0.07\}$. (B) Spearman correlations between $range_{AS}$ and sigmoid steepness parameter of WM learning trajectories (more positive suggests faster gains).
